# Managing institutional conflicts: Stakeholder accounts of communication between conflict of interest and technology transfer offices

**DOI:** 10.1371/journal.pone.0304519

**Published:** 2024-08-07

**Authors:** Matthew S. McCoy, Michaela Ward, Rebecca Neergaard, Steven Joffe, Julia E. Szymczak

**Affiliations:** 1 Department of Medical Ethics and Health Policy, Perelman School of Medicine, University of Pennsylvania, Philadelphia, Pennsylvania, United States of America; 2 Leonard Davis Institute of Health Economics, University of Pennsylvania, Philadelphia, Pennsylvania, United States of America; 3 Mixed Methods Research Lab, Perelman School of Medicine, University of Pennsylvania, Philadelphia, Pennsylvania, United States of America; 4 Department of Pediatrics, Perelman School of Medicine, University of Pennsylvania, Philadelphia, Pennsylvania, United States of America; 5 Division of Epidemiology, Department of Internal Medicine, University of Utah School of Medicine, Salt Lake City, Utah, United States of America; University of Agriculture Faisalabad, PAKISTAN

## Abstract

There have been repeated calls for academic institutions to develop policies and procedures to manage institutional conflicts of interest (ICOI) arising from technology transfer activities. While prior research has examined adoption of ICOI policies by medical schools and universities, little is known about how these institutions handle ICOI in practice, hindering the development of evidence-based recommendations to improve ICOI management. To address this gap, we conducted semi-structured interviews with 25 senior administrators responsible for research integrity and conflict of interest issues at academic institutions. Data were analyzed using a descriptive qualitative content analysis approach, combining flexible coding to index the interview data with close examination, interpretation, and synthesis of coded content. Participants identified communication and information sharing between conflict of interest (COI) and technology transfer (TT) offices as a critical factor in the effective management of ICOI and suggested several strategies to strengthen coordination between these offices. These findings suggest that academic research institutions could strengthen COI programs by taking measures to improve communication and information sharing between COI and TT offices.

## Introduction

Academic institutions are increasingly involved in commercializing research. This trend is especially notable in the biomedical research arena, where universities now stand alongside multinational corporations as leading recipients of life sciences patents [1, 2]. Among the largest research institutions participating in the Association of University Technology Managers’ (AUTM) annual survey, the average institution applied for 221 patents, executed 83 licenses and options, spun out more than 12 start-up companies, and earned over $40 million in licensing income in 2020 alone [3].

Technology transfer from the academic to the private sector is critical to ensuring that academic discoveries are translated into commercially available therapies and interventions that benefit patients and public health [4]. However, university involvement in research commercialization can also generate institutional conflicts of interest (ICOI), which grow in number and complexity as institutions expand their technology transfer activities.

ICOI refer to circumstances in which “an institution’s own financial interests or those of its senior officials pose risks of undue influence on decisions involving the institution’s primary interests” [5] In the context of technology transfer, ICOI arise because academic institutions’ financial interests in research commercialization may conflict with core institutional values. Prior research suggests that academic research institutions may, for example, encourage faculty to withhold or delay publication of scientific results while they acquire patent rights to intellectual property, or they may restrict access to intellectual property that they own, negatively affecting public health [6–8]. Additionally, case studies of research misconduct have raised concerns that institutional financial interests could compromise independent review and oversight of research conducted at the institution, undermining research integrity and risking harms to research participants [9–11]. In addition to their immediate impacts, ICOI could also jeopardize public trust in academic institutions [5].

In light of these concerns, public and private organizations including the Department of Health and Human Services (HHS), the National Academy of Medicine (formerly the Institute of Medicine), the American Association of Medical Colleges (AAMC), and the Associations of American Universities have called for research and policy development aimed at ensuring that academic institutions can effectively identify and manage ICOI related to their involvement in commercializing research [5, 12, 13].

Existing studies in this area have focused on formal policies and management structures for addressing ICOI. In a 2008 survey of United States (U.S.) schools of medicine, Ehringhaus and colleagues found that while a majority of institutions had implemented management structures that separate technology transfer from research oversight, including conflict of interest oversight, just 38% of institutions had a written policy addressing institutional financial interests [14]. A 2011 report issued by the Inspector General of HHS revealed that only 44% of NIH grantee institutions had written ICOI policies [12]. In a 2016 study, Resnik and colleagues found that only 28% of the top U.S. academic research institutions (by research funding) had a publicly available ICOI policy and that just 15% had adopted the AAMC recommendation of developing an ICOI committee [15]. Slaughter and colleagues analyzed ICOI policies of U.S. universities and found that they varied considerably in the activities they prohibited, the disclosure processes they required, and the tools they provided for the management of ICOI [6].

While highlighting important gaps and inconsistencies in the adoption of ICOI policies, a limitation of these studies is that they provide little information about how academic research institutions handle ICOI in practice. As Slaughter and colleagues suggest, ICOI policies are only as effective as the administrators and committees that implement them. It is possible that institutions with robust ICOI policies nonetheless face significant challenges in implementing those policies. Conversely, it is possible that institutions have developed best practices and standard operating procedures for addressing ICOI that are not reflected in written policies and hence not captured in published studies. Thus, there remains limited knowledge of the real-world complexities of ICOI management practices, a knowledge gap that hinders the development of evidence-based recommendations to more effectively manage ICOI that arise in technology transfer.

To address this gap, we conducted a qualitative study using semi-structured interviews with senior administrators responsible for research integrity and conflict of interest issues at universities, medical schools, and research hospitals. We sought a nuanced understanding of current approaches to ICOI management and stakeholder-informed accounts of their effectiveness. In this paper, we describe participants’ views of a critical but understudied aspect of ICOI management: communication and information sharing between conflict of interest (COI) and technology transfer (TT) offices.

TT offices play a critical part in academic institutions’ commercial activities by managing the patenting and licensing intellectual property and supporting the development of faculty led spin-out companies. Some TT office also take a broader role in cultivating and maintaining industry partnerships [16, 17]. In light of TT offices’ central role in activities that generate ICOI, the AAMC and Association of American Universities (AAU) recommended that TT offices provide COI offices with at least quarterly reports of commercial activities, thereby allowing COI offices to track institutional financial interests and appropriately manage resulting ICOI [13]. We sought to understand how these and other interactions between COI and TT offices play out in practice, what challenges they tend to encounter, and how they might be strengthened.

## Methods

### Study design, setting, and participants

We conducted a descriptive qualitative study [18] using semi-structured interview methods between February and May 2021. Eligible participants included administrators responsible for research integrity and COI issues at U.S. academic research institutions ranked in the top 75 nationally by research funding. We sampled participants from these institutions because the more research funding an institution has, the greater their TT activities [3]. Because prior research has shown that responsibility for research integrity and COI issues can reside in different offices across academic research institutions [12], we leveraged the AAMC Forum on Conflict of Interest in Academe (FOCI) to recruit eligible participants. FOCI serves as “a forum for the leadership in the biomedical arena who oversee and manage conflicts of interest to promote the highest ethical and professional standards in the conduct of their institutions” [19]. Members represent a wide range of U.S. universities, schools of medicine, and research hospitals, and either bear primary responsibility for research integrity and COI issues at their respective institutions or are knowledgeable about where these responsibilities reside. We recruited FOCI members via email to request an interview or referral to a more appropriate individual at the same institution—a recruitment strategy which has proven successful in previous studies of institutional approaches to conflict of interest [20]. We purposively sampled participants by funding status (public vs. private) to capture a range of perspectives across institution type.

### Data collection

Interviews were conducted using a semi-structured guide developed by the investigative team (see Supplemental Digital S1 Appendix for interview guide). The interview guide was designed to be open-ended, non-leading and to elicit detailed information about ICOI in practice—the interactions, processes, and real-world challenges of the work. Key domains in the guide include: 1) interactions between the COI and TT offices, 2) processes for identifying and managing ICOI in the TT process, 3) strengths and challenges in the management of ICOI in TT, and 4) suggestions for additional policy guidance. Interviews were conducted over the phone by the lead author (MSM) and audio recorded. Verbal informed consent was obtained and recorded prior to all interviews. The University of Pennsylvania Institutional Review Board determined that the study was exempt (#843481).

### Analysis

Audio recordings were transcribed, de-identified, and entered into NVivo for management and analysis. We utilized a descriptive qualitative content analysis approach, combining flexible coding to index the interview data with close examination, interpretation, and synthesis of the coded content [21, 22]. First, we performed a close reading of two transcripts to develop a codebook that included domains from the interview guide plus emergent themes identified during data collection. The codebook was reviewed by the investigator group, refined, and all codes defined. Second, two trained coders from the University of Pennsylvania’s Mixed Methods Research Lab (MW and RN) applied the codebook to all transcripts; inter-rater reliability queries (ĸ = .84 [range.75-.84]) were run periodically to ensure agreement and consistent application of codes. Third, the investigator group examined the content of individual codes across respondents to identify recurrent patterns in the data.

## Results

### Participants

Of 43 administrators recruited to participate in the study, 25 were interviewed, for an overall response rate of 58%. Table 1 describes characteristics of participating respondents.

**Table 1:** Characteristics of interview sample (N = 25).

Characteristic	No. of Respondents	% of Respondents
Institution type		
Public	11	44%
Private	14	56%
Respondent position type*		
COI office director/associate	12	48%
Research integrity/compliance officer	8	32%
COI committee chair/director	4	16%
Other	3	12%
Respondent years in current position		
1–5	11	44%
6–10	10	40%
11–15	2	8%
>16	1	4%
(unrecorded)	1	4%
Sex		
Male	11	44%
Female	14	56%

*Position types sum to more than 25 because some respondents held multiple positions.

### An “inherent conflict:” Tension between the aims of TT and COI offices

Across both public and private institutions, participants reported a growing emphasis on entrepreneurialism at their institutions. They associated this “entrepreneurial push” (Respondent [R] 18) with increased licensing activity, expanded support for faculty-led spin-out companies, and higher level of risk tolerance in institutional-industry partnerships.

Against this backdrop, participants reflected on what they perceived as a basic tension between the aims of TT and COI offices. They saw TT offices as focused primarily on advancing their institution’s entrepreneurial goals, which they contrasted with the COI office’s role in enforcing compliance with ICOI policies, ensuring research integrity, and protecting the institution’s reputation. Interviewees described this tension in terms of “competing priorities” (R12), “completely different interests” (R14), “cross purposes” (R9), or a “almost a natural opposition” (R6) between the two offices. As one participant stated, “their [the TT office’s] emphasis is focused on getting options and licenses and protecting intellectual property. My priority is protecting the institutional research for individual researchers” (R17). One participant expanded on the nature of the "struggle" between the aims of TT and COI offices:

We’re creating things to actually have them reach patients and advance the public good, we need to work with—we need to license them out to work with the for-profit sector. This is all normal and good but it is fascinating and a struggle to realize that their day-to-day activities are driven on getting the best deal and putting these technologies out and making money. And that inherent conflict with being part of a nonprofit institution is just something that I struggle with and being on the conflict of interest side, where then we’re reacting and managing.

(R19).

Many participants felt that they were able to have productive working relationships with colleagues in the TT office despite this tension. They explained that these productive working relationships were possible because of a shared sense that TT and COI offices played distinct but complementary roles in advancing the institution’s broader mission, explaining that the TT and COI offices were ultimately “on the same team” (R2) or “working under the same value system” (R9). One participant suggested that differing priorities of the TT and COI offices manifested in a “creative tension” that served the broader mission of the institution (R16).

Others, however, sensed that colleagues in the TT office and other institutional stakeholders failed to appreciate the role of the COI office, viewing it not as a partner, but as a drag on TT:

I think not just my technology transfer office, but most of my university perceives me as a rate limiting step where they don’t understand that it’s not just that I just stepped away from my desk and I’m refusing to push a button, but that the activities that we engage in my office are actually protecting individuals legally and reputationally.

(R17)

The perception of the COI office as a bureaucratic hurdle had consequences for communication between the COI and TT offices. As one participant noted:

I think we’re seen as a stop, and we’re still working to change the mindset to ‘we are here to help you,’ and we’re at stop right now because you loop us in once there’s an issue. If we were part of the design process or the brainstorming, we could just tell you what the rules are or the regulations, and then you could go from there without us stopping your process.

(R14)

Another participant reflected on personnel changes at the institution in the TT office, the conflict between corporate and academic norms, and the consequences of this tension for communication:

Yeah, so we had a group that we had brought in [to the TT office] to really push innovation and I think that the idea was to hire a lot of corporate people who had the other side of the picture in innovation. And so they brought a lot of those folks in and it really was, it did, that model just was not working well. So now they’ve kind of pulled back and they’ve gone back to the idea of, you know individuals who are more academically focused and more research focused. I think there was no appreciation for the gravity of human subject research with the corporate people that were being hired to manage our [TT] office in the last few years, yeah. It felt like the wild-wild west, and nobody was communicating and everybody’s out there kind of doing their moving and shaking and not really paying attention to what they were doing.

(R21)

Notably, several participants said that while they had initially had challenging relationships with colleagues in the TT office, they had been able to strengthen those relationships over time. They largely attributed these improved relationships to the development of trust and mutual understanding between the two offices. One trust-building strategy was to be more transparent about the COI office’s own priorities and processes:

I think it was really important to have them sit in on some of our discussions to hear what we think about when we look at some of these situations for them to really understand, oh, I get it. I get we’re trying to protect the science and the integrity of this research. And when they hear that, it really resonates with them and then that can be taken back to their shop.

(R9)

While some participants found that they had been able to work directly with TT colleagues to improve collaboration between offices, others noted that they had relied or would need to rely on senior leadership such as Vice Provosts (VP) for Research to foster a productive relationship:

We have a meeting next week with [the TT office], and the VP for Research will be present, to try to address what we’re doing and what we are trying to protect the university from. And then to see if, this is kind of our test case—to see if that will spark a willingness on their part to loop us in a little bit more than they currently do.

(R14)

Another participant described collaboration between the COI and TT offices being challenging prior to the arrival of a VP for Research who “took it upon themselves to say conflicts of interest, including institutional conflicts of interest, are a huge concern and should be addressed more broadly at the institution” (R25).

### “It comes down to communication:” Timing, formalization, integration and automation

Participants at both public and private institutions consistently emphasized that communication and exchange of information with their institution’s TT office was essential to their ability to manage ICOI effectively. When asked to reflect on the strengths of their institution’s approach to ICOI, those who felt that their programs were generally successful often traced their strengths to good communication with the TT office:

I think honestly it comes down to communication…Our policies aren’t the best policy you’re going to see. Our processes aren’t the best process you’re going to see. You’ll find others with stronger, more rigorous programs and more sophisticated thinking than [University] on some of this. But we have really good communication with our tech transfer office and we’re good partners. And that fills some of those gaps.

(R2)

When prompted to reflect on ways in which their institution’s approach to managing ICOI could be strengthened, participants indicated a desire to improve communication with their TT office far more often than they spoke about improving policies or committee processes. Even participants who felt that they had strong relationships with colleagues in the TT office suggested that strengthening communication and information sharing between the offices would improve the management of ICOI.

While participants offered a number of ideas for improving communication between the COI and TT offices, their suggestions highlighted the importance of three dimensions of information sharing that they viewed as especially critical: timing, formalization, and integration and automation.

#### Timing

A number of participants stressed that it was important for COI offices to be notified of TT agreements and any research involving the institution’s intellectual property “earlier rather than later” (R21). Participants suggested different standards of “early” communication and information sharing related to TT and research. With respect to licensing deals, participants explained that they would like for the COI office to be informed of deals around the time they are executed, though some thought that there were scenarios in which the TT office should consult the COI office earlier in the process. Early communication is critical, they noted, because it allows the COI office and relevant committees to apply an appropriate management plan to research utilizing licensed technology, rather than “scrambling behind someone saying, ‘great, you have this terrific thing taking off, and now I’m coming along and telling you all the things you can and cannot do.’” (R21). Participants noted that when their offices are not involved early it can lead to delayed research and other challenges:

We would find out post-hoc once a sponsored research agreement or a clinical trial was booked and the investigator was on it, which is really too late. It’s too late to modify the research or modify the investigator’s role in the research. It’s not too late, but it’s certainly more cumbersome and it delays the research.

(R3)

To facilitate early communication between COI and TT stakeholders, some participants reported that their institution required the COI office or a COI committee to sign off on licensing deals before they could be executed. In these cases, the committee or COI office did not typically advise the TT office on the business aspects of the license, but the process did ensure that COI officers were sufficiently informed:

Our policy requires the technology transfer…to have approval from the conflict of interest committee…before a licensed startup company is created, and so they’re always coming to us…The committee will learn who are the faculty members or staff that are involved in the licensed startup company, what positions might they take in the licensed startup company, what equity are they taking equity or not, and also whether or not the university is taking equity.

(R7)

While some participants expressed a desire to implement such a policy, others thought that requiring prospective sign off for typical licensing deals would be unnecessary because there was rarely variation in the nature of the agreements.

#### Formalization

To facilitate improved communication, several participants expressed a desire for standard operating procedures that establish formal protocols for information sharing between the TT and COI office. Some participants who perceived TT offices as reluctant or slow to share information with the COI office attributed these challenges to the lack of formal policies establishing expectations for communication and information sharing:

I would have to say at [University] the challenge has been different offices operating in silos, of not having the communication of not having the policy to point to okay, this is in our books

(R25).

Closely related to concerns about the irregular pace of communication, some participants suggested that a lack of formal protocols for information sharing created uncertainty about the comprehensiveness of reports received from TT offices. As one noted, “the information I get is good, but I don’t know what information I’m not getting” (R14).

Even where participants felt that they had strong informal communication with colleagues working in TT, many reflected on challenges of keeping track of irregular invention disclosures from investigators and licensing officers in the TT office—each of whom “may have had their own processes” for communicating with the COI office (R6)—and the need for establishing “very defined communication channels.” (R11). As one participant explained, instituting standard processes for information sharing is a goal for the future:

We aren’t as systematic as one might be. One of the things we’re discussing is building in standard processes for informing one another…Right now, it is informal, you know, I call the director, the director calls me. We’re certainly on good terms. We try to keep each other in the loop but I think, you know, one of our tasks going forward is to better systematize that role

(R16).

#### Integration and automation

Many participants reported that lack of access to TT databases and information systems inhibited their ability to effectively manage ICOI and suggested that better data integration would improve communication between TT and COI offices. Participants described the need for resources like shared database that would allow COI offices to directly obtain information about innovation disclosures, patents, licensing deals, and other commercial arrangements. Participants explained that such resources would reduce reliance on TT officers, institutional administrators, and investigators to report the details of commercial arrangements to the COI office and mitigate the risk the institutional interests would be overlooked. A participant from an institution that had developed a shared database described it as a “failsafe,” noting that the COI office used the database to run monthly reports “to make sure we’ve caught any new license agreements in case someone forgets to copy us” (R24).

Closely related to the desire for integrated information systems, several participants noted that they would like to see less reliance on manual information exchanges, which they saw as prone to error and delays, and greater use of automated systems that “take some of the human component out of it” (R18). One participant described that their office learned of new licensing deals through a quarterly report from the TT office, and pointed out that if the list was late or incomplete, “there could have been companies that license agreements were done [with] and we don’t flag it in our system” (R8). The same participant described ongoing efforts to “get away from a person having to send us an Excel file for us to upload stuff into our system” to an automated approach in which new patents and license agreements “flags all of the systems everywhere” (R8). Expressing a similar desire, another participant explained that “ideally, you’d have a real time feed into your system with a notification [when] a new license popped in: here’s the license, here are the terms, that sort of thing” (R5).

## Discussion

In this qualitative study of administrators’ views about their institutions’ approach to handling ICOI, we found that participants consistently identified communication and information sharing between COI and TT offices as a vital factor in the effective management of ICOI. Participants’ perceptions of the strengthens and limitations of their institutions’ ICOI programs did not vary significantly by institution type (public vs. private). However, participants who felt that they communicating effectively with counterparts in the TT office tended to view their ICOI programs as effective.

Our analysis revealed several specific dimensions of communication and information sharing between COI and TT offices that can influence administrators’ ability to identify and manage ICOI, including timing, formalization, and integration and automation. Participants suggested several strategies to improve communication between COI and TT offices. These strategies included ensuring that COI and TT offices had mutual understanding and appreciation of each other’s goals and processes; promoting interaction between COI and TT offices early in the technology transfer process; developing formal reporting procedures; leveraging shared databases; and automating information exchange to reduce error.

Our study offers lessons for academic institutions seeking to strengthen their COI programs. Despite widespread agreement that academic institutions should improve their handing of ICOI, there remains limited guidance for administrators wishing to make progress in this area. Notably, the United States Public Health Service’s (PHS) COI rules, which establish standards for dealing with *individual* COI, do not address *institutional* COI [23]. Our findings suggest that even in the absence of authoritative guidance, academic research institutions might be able to immediately strengthen their COI programs by taking measures to improve communication and information sharing between COI and TT offices. Measures might include investing in database infrastructure and developing standard operating procedures to enable efficient and comprehensive information sharing. Our findings also suggest that broader efforts to foster rapport and a sense of shared purpose between COI and TT offices may indirectly but significantly improve administration of ICOI-related matters. Senior research officials can play a pivotal role in leading these efforts and ensuring the COI and TT offices are operating under a shared value framework.

Results of this study can also inform and reinforce guidance about information sharing between COI offices and other institutional offices. A model ICOI policy published by AAMC and AAU in 2008 recommends that TT offices provide *at least* quarterly reports to COI regarding licensing arrangements, patents, and invention disclosures. Our findings suggest that in an era of increasing research commercialization, quarterly reporting may be insufficient for institutions with a high volume of technology transfer and that more frequent or even real time information sharing may be appropriate. The model policy also recommends that COI offices’ tracking of licensing agreements, as well as gifts and sponsored research agreements, would be “greatly facilitated by the development of one or more comprehensive databases by the institution.” Our analysis affirmed the value of shared databases while also revealing that more than 15 years after the AAMC and AAU issued this recommendation, many institutions lack such resources.

This study has limitations. First, we are limited in our ability to generalize our findings beyond the settings we studied. Second, the relatively small size of our sample may have prevented us from significant variation between public and private institutions or other subgroups in our study. Third, while our approach to sampling interview participants was informed by previous research suggesting the distributed nature of responsibility for ICOI, it is possible that we omitted important stakeholders, leading to bias in our sample. Despite these limitations, this study represents an important early step in generating knowledge about institutional dynamics that characterize ICOI management and supports recent calls for further study of ICOI at academic research institutions [24, 25]. Future research utilizing survey methods, informed by these results, could capture a broader range of stakeholder perspectives, including those of TT officers, and permit further exploration of both commonalities and variation across institutions.

## Conclusion

This study makes a significant contribution to existing work addressing the administration of ICOI-related matters at academic research institutions. While prior studies address the adoption and provisions of ICOI policies, we used a qualitative approach to better understand real world challenges faced by those responsible for ICOI-related matters at their institutions. Even where institutions have ICOI policies in place, one of the greatest challenges that COI officers face is ensuring that they have timely access to information regarding their institution’s commercial activities. As the volume of technology transfer at academic research institutions continues to increase, there is a critical need to address this challenge.

## Supporting information

S1 Appendix Interview guide. (DOCX)
