# Correction: Managing institutional conflicts: Stakeholder accounts of communication between conflict of interest and technology transfer offices

**DOI:** 10.1371/journal.pone.0313873

**Published:** 2024-11-13

**Authors:** The PLOS ONE Staff

There are errors in the Data Availability, Funding, and Competing Interests statements. The publisher apologizes for these errors.

The correct Data Availability statement is: All quantitative data are included in Table 1. Conclusions reached in the article are based on de-identified excerpts of qualitative data included in the article. Due to the small sample size and potentially sensitive nature of the qualitative data collected for this study, interview transcripts are not available in line with our agreement with the University of Pennsylvania Institutional Review Board.

The correct Funding Statement is: This work was supported by the National Center for Advancing Translational Sciences [administrative supplement to 5UL1TR001878]. The funders had no role in study design, data collection and analysis, decision to publish, or preparation of the article. No additional external funding was received for this study.

The correct Competing Interests statement is: I have read the journal’s policy, and the authors of this article have the following competing interests: Dr. Joffe is a paid member of a Data Safety and Monitoring Board for CSL Behring.
