# Supplementary material for: Managing institutional conflicts: Stakeholder accounts of communication between conflict of interest and technology transfer offices
Source: PLoS One. 2024 Aug 7;19(8):e0304519. doi: 10.1371/journal.pone.0304519 (PMC11305534; doi:10.1371/journal.pone.0304519)
Supplement: S1 Appendix — (DOCX) [file pone.0304519.s001.docx]

**Guide for semi-structured interviews**

- Thank you for agreeing to participate in this project.
- The interview should take about 45 minutes
- Our aim to gain a better understanding of the ways in which academic research institutions currently manage **Institutional** COI in **technology transfer** and how current approaches might be strengthened.
- We’re speaking to administrators responsible for conflict of interest issues at academic research institutions. We hope the interviews will provide evidence of common challenges, existing policy gaps, and promising approaches that will inform future policy.
- Your participation in the study is completely voluntary. You are free not to answer any questions that you feel uncomfortable about, and you are welcome to pause or end the interview at any time.
- Your name, your institution, and any other personally identifying information will not be used, and you will be assigned a de-identified code in all study materials.
- Do you consent to this interview?
- Do I have your permission to audio record the interview?

**Background**

1. What is your position at [institution]?
2. How long have you been in this position?
3. Can you describe your main responsibilities?

**Technology transfer process and challenges**

1. What are your specific responsibilities with respect to technology transfer agreements?
2. Can you describe the way you interact with your institution’s technology transfer office?
   1. Are there any aspects of these interactions that are particularly challenging?
   2. Or that work particularly well?
3. At your institution, what sorts of arrangements are taken to constitute ICOI
   1. Equity, IP with licensing or royalty arrangements, other?

Do ICOI considerations extend to when leaders (deans, dept chairs, etc) have financial relationships with relevant companies, or are they limited to when the institution itself has a stake?

If yes to the above, how do you know when an institutional leader has a stake?

1. Who is responsible for reviewing technology transfer agreements for ICOI issues?
   1. Is there a standing committee?
      1. General COI committee or special ICOI committee?
      2. To whom does it report?
      3. What kinds of actions can the committee take?
   2. What triggers an ICOI review?
      1. Is there prospective review of licensing and equity agreements?
2. Does your institution have an ICOI policy?
   1. If not, why do you think your institution does not have an ICOI policy?
   2. If so, how well does it help you navigate the challenges you described
   3. Are there ways that the policy might be updated or revised so that it would be more useful in this regard?
   4. Are there industry interactions that aren’t capture by ICOI policies that perhaps could be?
3. What do you see as the main challenges related to identifying and managing ICOI in the technology transfer process?
   1. Have any of these challenges changed or intensified over time?
   2. Looking down the road, what do you see as emerging challenges in this area?
4. What do you see as the main strengths of your institution’s approach for managing ICOI in technology transfer?
   1. Have you developed any best practices that you think other institutions might follow?
5. How, if at all, could your institution’s approach be improved?
6. Are there any areas related to ICOI in technology transfer where you think that additional guidance from a body like the AAMC or AAU would be helpful?
7. Is there anything else you’d like me to know?
